# Pioglitazone restores mitochondrial function but does not spare cortical tissue following mild brain contusion

**DOI:** 10.1093/braincomms/fcad032

**Published:** 2023-02-13

**Authors:** W Brad Hubbard, Hemendra J Vekaria, Olivia J Kalimon, Malinda L Spry, Emily P Brown, Todd J Kilbaugh, Patrick G Sullivan

**Affiliations:** Lexington Veterans’ Affairs Healthcare System, Lexington, KY 40502, USA; Department of Physiology, University of Kentucky, Lexington, KY 40536, USA; Spinal Cord and Brain Injury Research Center, University of Kentucky, Lexington, KY 40536, USA; Spinal Cord and Brain Injury Research Center, University of Kentucky, Lexington, KY 40536, USA; Spinal Cord and Brain Injury Research Center, University of Kentucky, Lexington, KY 40536, USA; Department of Neuroscience, University of Kentucky, Lexington, KY 40536, USA; Spinal Cord and Brain Injury Research Center, University of Kentucky, Lexington, KY 40536, USA; Spinal Cord and Brain Injury Research Center, University of Kentucky, Lexington, KY 40536, USA; Department of Anesthesiology and Critical Care Medicine, Children’s Hospital of Philadelphia, Perelman School of Medicine, University of Pennsylvania, Philadelphia, PA 19104, USA; Lexington Veterans’ Affairs Healthcare System, Lexington, KY 40502, USA; Spinal Cord and Brain Injury Research Center, University of Kentucky, Lexington, KY 40536, USA; Department of Neuroscience, University of Kentucky, Lexington, KY 40536, USA

**Keywords:** traumatic brain injury, bioenergetics, mitoNEET, oxidative stress, TBI

## Abstract

Pioglitazone interacts through the mitochondrial protein mitoNEET to improve brain bioenergetics following traumatic brain injury. To provide broader evidence regarding the therapeutic effects of pioglitazone after traumatic brain injury, the current study is focused on immediate and delayed therapy in a model of mild brain contusion. To assess pioglitazone therapy on mitochondrial bioenergetics in cortex and hippocampus, we use a technique to isolate subpopulations of total, glia-enriched and synaptic mitochondria. Pioglitazone treatment was initially administered at either 0.25, 3, 12 or 24 h following mild controlled cortical impact. At 48 h post-injury, ipsilateral cortex and hippocampus were dissected and mitochondrial fractions were isolated. Maximal mitochondrial respiration injury-induced deficits were observed in total and synaptic fractions, and 0.25 h pioglitazone treatment following mild controlled cortical impact was able to restore respiration to sham levels. While there are no injury-induced deficits in hippocampal fractions, we do find that 3 h pioglitazone treatment after mild controlled cortical impact can significantly increase maximal mitochondrial bioenergetics compared to vehicle-treated mild controlled cortical impact group. However, delayed pioglitazone treatment initiated at either 3 or 24 h after mild brain contusion does not improve spared cortical tissue. We demonstrate that synaptic mitochondrial deficits following mild focal brain contusion can be restored with early initiation of pioglitazone treatment. Further investigation is needed to determine functional improvements with pioglitazone beyond that of overt cortical tissue sparing following mild contusion traumatic brain injury.

## Teaser

Hubbard *et al*. report that a workflow to isolate total, glia-enriched, and synaptic mitochondrial subpopulations demonstrates that synaptic mitochondrial changes are apparent following traumatic brain injury in mice. Early pioglitazone administration can improve mitochondrial bioenergetics following mild brain contusion, although these improvements do not translate to overt tissue sparing.

## Graphical Abstract

**Graphical Abstract:**
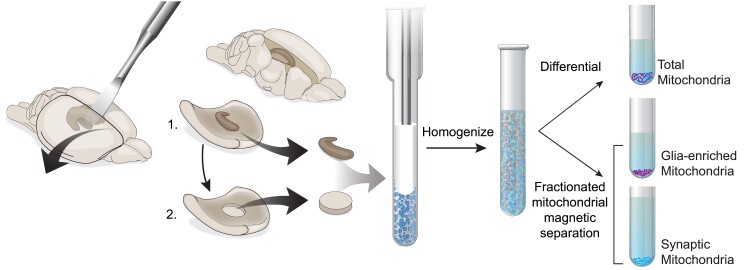

## Introduction

Traumatic brain injury (TBI) continues to affect numerous individuals in the USA who suffer from the on-going consequences of post-TBI symptomatology.^1^ As such, there are no Food and Drug Administration-approved therapeutics for TBI to alleviate on-going neurological disorders. An important hallmark and target of TBI pathophysiology is mitochondrial dysfunction. Mitochondria have numerous cellular functions, including ATP production, calcium buffering, and maintaining oxidant homeostasis. Importantly, protein signalling within mitochondria can regulate function and respiration. One such protein, mitoNEET, has been identified as an outer mitochondria membrane protein that can tightly regulate respiratory capacity, operating as a ‘power switch’ of mitochondrial function.^2,3^ Importantly, ligands to mitoNEET, including pioglitazone, have proven efficacious in models of neurotrauma.^4–10^ Even with the published evidence, no study to date has assessed pioglitazone treatment as mitochondrial-direct therapy in a mild severity model of brain contusion.

Our group has streamlined techniques to examine profiles of mitochondrial respiration from synaptic and non-synaptic (or glia-enriched) fractions.^11^ In this study, we perform side-by-side comparison of total, glia-enriched, and synaptic respirometry from unique brain regions following traumatic brain injury. This study dovetails with our previous publication,^4^ which found that pioglitazone could target mitochondrial dysfunction following severe preclinical TBI, and contributes to the alleviation of on-going neurobehavioral disruption and cortical damage. This study was performed to increase efforts toward rigour and reproducibility in neurotrauma research by examining therapeutic efficacy in a mild model of focal brain contusion injury. We used a mild severity controlled cortical impact (CCI) to assess pioglitazone therapy on mitochondrial subpopulation bioenergetic analysis and neuroprotection.

## Materials and methods

### Experimental design

All of the studies performed were approved by the University of Kentucky Institutional Animal Care and Use Committee, in compliance with the guidelines of the Association for the Assessment and Accreditation for Laboratory Animal Care, International and the National Institutes of Health Guide for the Care and Use of Laboratory Animals.^12^ Animal experiments complied with Animal Research: Reporting of In Vivo Experiments guidelines. All experiments were conducted using male C57BL/6J mice (2–3 months old; Jackson Laboratories, Bar Harbor, ME, USA).

The animals were housed five per cage, maintained in a 14 h light/10 h dark cycle, fed a balanced diet *ad libitum*. Animals were randomly assigned to groups, based on injury designation (sham or CCI) and treatment designation (vehicle or pioglitazone). Treatments were given in random order. All experimentation was performed blinded to treatment groups. For all outcomes, experiments were conducted with biological replicates of *N* = 4–8/group. Additionally, technical triplets were used in each assay.

Two separate cohorts were used for this study. The acute study was conducted to examine the effect of pioglitazone treatment after mild brain contusion on total, glia-enriched, and synaptic mitochondrial bioenergetic measures in the brain. Experimental groups for the acute study were euthanized at 48 h after injury. The subacute efficacy study was conducted to examine how modulation of acute mitochondrial bioenergetics with pioglitazone translated into cortical neuroprotection after mild brain contusion. Experimental groups for the subacute efficacy study were euthanized at 15 days after injury. Mice received a bolus (i.p.) administration of pioglitazone (100 µL volume; 1:1 DMSO and PEG400) at either 0.25, 3, 12, or 24 h after mild CCI, based on the treatment strategy outlined in Hubbard *et al*.^4^ The 15-day efficacy study utilized 7-day duration Alzet Mini Pump 1007D (release of 0.5 µL/h; Alzet, Cupertino, CA, USA).

### Controlled cortical impact

The CCI procedure was performed according to past studies with some modifications.^4,13^ Importantly, our model was modified to a mild CCI, incorporating cortical depression depth of 0.5 mm with confined tissue loss to the cortical region.^14^ Briefly, anaesthetized (2.5% isoflurane) mice were fixed with ear bars in a stereotaxic frame. After scalp incision, a 3 mm craniotomy was performed lateral to midline and mice received a pneumatic impact (TBI-0310 Impactor, Precision Systems and Instrumentation, Fairfax, VA, USA: 0.5 mm depth; 3.5 m/s velocity; 500 ms dwell) using a 2 mm impactor tip directly to the brain with dura intact. Sham animals received a craniotomy but had no impact. Following impact, the craniotomy was covered with absorbable haemostat (Surgicel), and skin clips were used to close the wound. The CCI surgical and anaesthesia procedures were approximately 15 minutes for both sham and injured mice. Mice recovered on a heating pad until the animals were fully responsive.

### Total mitochondrial isolation

Mitochondria were isolated using previously employed differential mitochondrial isolation methods.^4,15^ Mice were asphyxiated with CO_2_, and the brain was dissected in mitochondrial isolation buffer, [215 mM mannitol, 75 mM sucrose, 0.1% bovine serum albumin, 1 mM ethylene glycol tetraacetic acid, and 20 mM 4-(2-hydroxyethyl)-1-piperazineethanesulphonic acid at pH 7.2]. Homogenized ipsilateral cortex (4 mm tissue punch around lesion site) and ipsilateral hippocampus underwent a series of centrifugations^13,15^ before synaptosomal mitochondria release by nitrogen depressurization.^16^ Mitochondrial pellets were resuspended in isolation buffer (without ethylene glycol tetraacetic acid), and protein concentration was measured with bicinchoninic acid protein assay kit (Pierce, Cat # 23227) at 560 nm absorbance on Biotek Synergy HT plate reader (Winooski, VT, USA).

### Glia-enriched and synaptic mitochondrial isolation

For a detailed breakdown of the fractionated mitochondrial magnetic separation technique, see Hubbard *et al*.^11^ Briefly, the remaining 1.5 mL of homogenate (described above) from ipsilateral cortical tissue and ipsilateral hippocampus was taken, and combined supernatants from the first and second low-speed spins were incubated for 30 min with anti-Tom22 microbeads (Miltenyi Biotec) at a concentration of 4 μL/1 mg wet tissue weight. The mixture was then added to magnetic-activated cell sorting separation LS Columns, attached to QuadroMACS™ Separator (Miltenyi Biotec, cat. no. 130-097-040), to capture free mitochondria (glia-enriched fraction). The eluate was collected for subsequent use. The columns were plunged causing magnetically attached non-synaptic mitochondria to dissociate and enter into the 15 mL conical tube. The glia-enriched samples were centrifuged and protein was analysed. The eluate sample was centrifuged to collect synaptosomes, and the synaptic mitochondria were released using pressurized nitrogen cell disruptor. The resulting solution was incubated with anti-Tom22 microbeads (1 μL/1 mg wet tissue) for 30 min. All sequential steps were performed as previously described.

### Measurement of mitochondrial bioenergetics

Mitochondrial bioenergetics were assayed in isolated mitochondria according to previous studies.^4,11,17^ Briefly, Seahorse XFe96 Flux Analyzer (Agilent Technologies, Santa Clara, CA, USA) was used to determine oxygen consumption rates (OCRs) in the presence of mitochondrial substrates, inhibitors, and uncouplers. On the day before the assay, the sensor cartridge was hydrated. On the day of the assay, injection ports A–D of the sensor cartridge were loaded to measure OCR in various respiration states. Chemical stocks were diluted appropriately in respiration buffer [125 mM KCl, 0.1% bovine serum albumin, 20 mM 4-(2-hydroxyethyl)-1-piperazineethanesulphonic acid, 2 mM MgCl_2_, and 2.5 mM KH_2_PO_4_, adjusted pH 7.2] to make the final concentration of the chemicals to 5 mM pyruvate, 2.5 mM malate, and 1 mM ADP (via Port A; State III_C1_), 2.5 µM oligomycin A (via Port B; State IV), 4 µM FCCP (via Port C; State V_C1_), and 1 µM rotenone and 10 mM succinate (via Port D; State V_CII_). Six-microgram total mitochondria, 3 µg non-synaptic, and 6 µg synaptic mitochondrial protein were loaded per well in a volume of 30 µL. Plates were centrifuged, and respiration buffer was gently added for total volume of 175 µL in each XFe96 well. OCRs were measured based on additions in each injection port. Due to multiple cohorts and Seahorse plates utilized, OCR values were analysed as per cent sham values. Sham groups were included on each of the four separate Seahorse plates, and OCR values were normalized to the average sham value on the same Seahorse plate.

### Protein carbonyl quantification

Mitochondrial homogenate aliquots (unused during respiration assays) were stored at −20°C until utilization for oxidative stress slot blots. Protein carbonyls (PC) were assessed in these samples as previously described.^15^ Protein concentrations were determined using a bicinchoninic acid protein assay. Polyclonal RbxDNP (from Oxy Blot Protein Oxidation Kit, Chemicon-Millipore, Billerica, MA, USA, Dilution 1:200; Cat No. S7150) was used for immunodetection. The membranes were scanned with a photo scanner (Epson Perfection V600, Long Beach, CA, USA), and slot-blot line densities were quantified by the ImageQuant TL software package (GE Healthcare Bio-Sciences, Piscataway, NJ, USA).

### Brain histology

One cohort of mice was euthanized at 15 days post-injury by intraperitoneal injection of Fatal Plus (Vortech Pharmaceuticals, Dearborn, MI, USA). Transcardial perfusion and cresyl violet staining were carried out as previously detailed.^4^ Briefly, mice were perfused with saline and 4% paraformaldehyde (PFA) before brain removal and post-fixation in 4% paraformaldehyde for 24 h. After sucrose cryoprotection, brain tissue was cut into 35 μm thick coronal sections and coronal tissue sections spaced ∼400 μm apart were mounted and stained with cresyl violet. Quantitative assessment of cortical damage was done with blinded, unbiased tracing protocol using HALO quantitative tissue analysis software (Indica Labs, Albuquerque, NM, USA). Data were converted to percentage of the contralateral cortex area, which was used as an internal control for each animal.

### Statistical analysis

Power analysis was conducted (using G*Power statistical software; version 3.0.10) for all experimental data and based on previous published literature from our group.^4,15^ Analysis was completed based on the analysis of variance (ANOVA) statistical tests and output of *F* score. *A priori* analysis was performed and effect size was calculated based on 10% data variance. Sample size was calculated for mitochondrial experiments using the following parameters: *α* = 0.05, 1 − *β* = 0.8, and standard deviation 10% of mean (effect size = 0.9) for experimental groups. There were no animals excluded from this study. All Seahorse biological replicate data were examined for standard quality control and were used. Statistical analysis was performed using Graph Pad Prism 8 (GraphPad Software, CA, USA). For all analyses, the significance of differences was set at *P* < 0.05. For mitochondrial and oxidative assays, one-way ANOVA was performed followed by a Dunnett’s *post hoc* test with groups compared to injured vehicle, when appropriate. For tissue sparing, one-way ANOVA was performed followed by a Tukey’s *post hoc* test.

## Results

**Figure 1:**
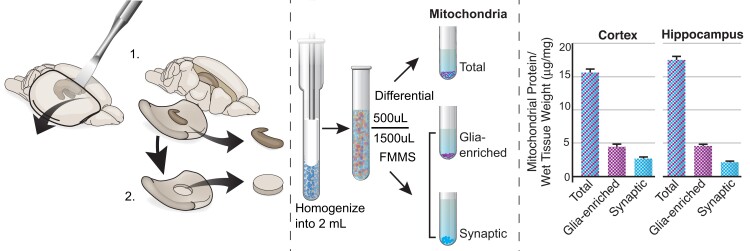
**Schematic of the technical workflow and protein output.** (Left) Dissection of ipsilateral hippocampus (top) and cortical punch (bottom) from a rodent model. (Middle) Homogenization of tissue resulting in fee-floating mitochondrial (glia-enriched) and synaptosomes. We utilized two protocols (differential and fractionated magnetic mitochondrial isolation) to derive three distinct mitochondrial subpopulations from the same sample. (Right) Mitochondrial protein output from three distinct populations from both the cortex and hippocampus. Protein was measured by bicinchoninic acid assay. Protein/wet tissue weight represented as mean ± SEM. Illustration by Matt Hazzard, University of Kentucky, Medical Illustration.

Based on the starting amount of wet brain tissue weight, we have measured the amount of protein in our mitochondrial populations using both differential isolation, to extract total mitochondria, and fractionated mitochondrial magnetic separation, to isolate glia-enriched and synaptic subfractions. We found that refined mitochondrial subpopulations greatly reduce the amount of protein compared to total mitochondria, as expected (Fig. 1). Glia-enriched mitochondrial fraction made up two to three times the amount of protein as compared to synaptic mitochondrial fractions in cortex and hippocampus.

**Figure 2:**
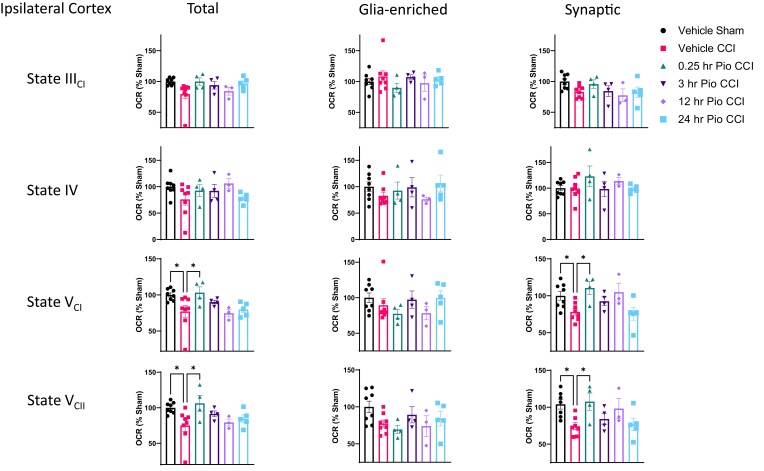
**Mitochondrial bioenergetics from ipsilateral cortex following mild brain contusion and subsequent pioglitazone administration.** Mice received either sham surgery or CCI followed by pioglitazone (20 mg/kg/day) initiated at either 0.25, 3, 12, or 24 h post-injury. An osmotic pump was inserted to ensure delivery of 20 mg/kg/day; mitochondria were then isolated from the ipsilateral cortex at 48 h post-injury, and bioenergetics were assayed using the Seahorse technology. There were no significant changes in State III_CI_ or State IV mitochondrial bioenergetics between groups in any subpopulation. However, there was a significant increase in State V_CI_ respiration in vehicle sham and 0.25 h pioglitazone (Pio) CCI groups compared to vehicle CCI in total mitochondria. One-way ANOVA, compared to vehicle CCI, Dunnett’s *post hoc*. *P < 0.037. *F*_5,26_ = 3.525. State V Complex I-mediated maximal mitochondrial respiration was unaltered in glia-enriched mitochondria. In synaptic mitochondria, there was a significant increase in State V_CI_ respiration in vehicle sham and 0.25 h Pio CCI groups compared to vehicle CCI. One-way ANOVA, compared to vehicle CCI, Dunnett’s *post hoc*. *P < 0.049. *F*_5,26_ = 4.14. There was a significant increase in State V_CII_ respiration in vehicle sham and 0.25 h Pio CCI groups compared to vehicle CCI in total mitochondria. One-way ANOVA, compared to vehicle CCI, Dunnett’s *post hoc*. *P < 0.022. *F*_5,26_ = 3.15. State V Complex II-mediated maximal mitochondrial respiration was unaltered in glia-enriched mitochondria. In synaptic mitochondria, there was a significant increase in State V_CII_ respiration in vehicle sham and 0.25 h Pio CCI groups compared to vehicle CCI. One-way ANOVA, compared to vehicle CCI, Dunnett’s *post hoc*. *P < 0.029. *F*_5,26_ = 3.48. Per cent OCR values represented as mean ± SEM with individual data points, *N* = 4–8/group. Data presented as percentage of sham.

We found that dysfunction in maximal mitochondrial respiration, mediated through both Complex I and Complex II, from cortical total mitochondria is aligned with deficits in synaptic mitochondrial function following mild CCI (Fig. 2). Further, pioglitazone treatment at 0.25 h post-injury can restore injury deficits in both total and synaptic populations of cortical mitochondria. There are no significant changes in mitochondrial respiration of glia-enriched cortical mitochondria following mild CCI.

**Figure 3:**
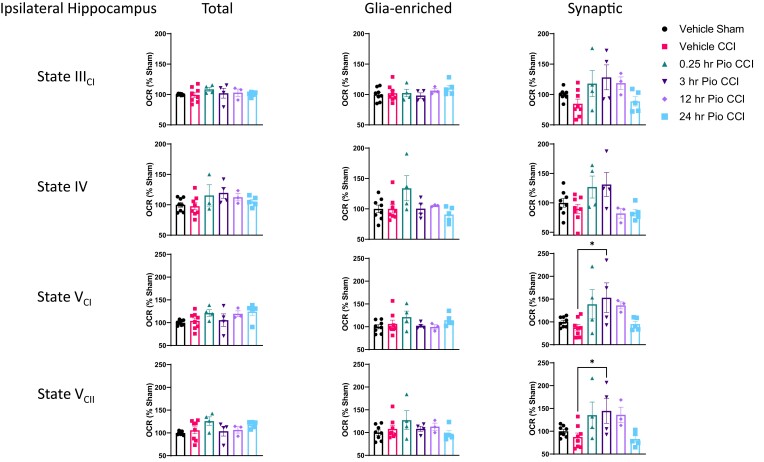
**Mitochondrial bioenergetics from ipsilateral hippocampus following mild brain contusion and subsequent pioglitazone administration.** Mice received either sham surgery or CCI followed by pioglitazone (20 mg/kg/day) initiated at either 0.25, 3, 12, or 24 h post-injury. An osmotic pump was inserted to ensure delivery of 20 mg/kg/day; mitochondria were then isolated from the ipsilateral hippocampus at 48 h post-injury, and bioenergetics were assayed using the Seahorse technology. There were no significant changes in State III_CI_ or State IV mitochondrial bioenergetics between groups in any subpopulation. There also were no changes in State V_CI_ or State V_CII_ respiration between groups for total and glia-enriched fractions. In synaptic mitochondria, there was a significant increase in State V_CI_ respiration in 3 h Pio CCI group compared to vehicle CCI. One-way ANOVA, compared to vehicle CCI, Dunnett’s *post hoc*. *P = 0.017. *F*_5,26_ = 3.31. There was also a significant increase in State V_CII_ respiration in 3 h Pio CCI group compared to vehicle CCI in synaptic mitochondria. One-way ANOVA, compared to vehicle CCI, Dunnett’s *post hoc*. *P = 0.031. *F*_5,26_ = 3.46. Per cent OCR values represented as mean ± SEM with individual data points, *N* = 4–8/group. Data were presented as percentage of sham.

We observe a lack of changes in mitochondrial respiration from total hippocampal mitochondria (Fig. 3). There are also no significant changes in mitochondrial respiration of glia-enriched hippocampal mitochondria following mild CCI. However, we show delayed 3 h pioglitazone-mediated increases in maximal mitochondrial respiration, mediated through both Complex I and Complex II, compared to vehicle CCI group in synaptic hippocampal mitochondria.

**Figure 4:**
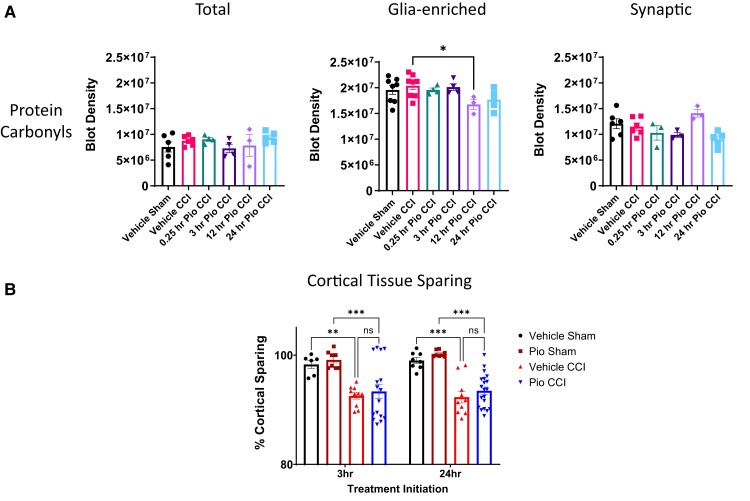
**Pioglitazone has modest effect on oxidative markers and does not alter cortical tissue sparing.** (**A**) Protein carbonyl levels in the cortex following mild brain contusion and subsequent pioglitazone administration. There were no changes in protein carbonyl (PC) levels between groups for total or synaptic mitochondrial subpopulations. Pioglitazone at 12 h administration had significantly lower PC as compared to vehicle CCI for glia-enriched mitochondria. One-way ANOVA, Dunnett’s *post hoc* compared to vehicle CCI. Total; *F*_5,20_ = 0.97. Glia-enriched; *P < 0.05, *F*_5,24_ = 2.74. Synaptic *F*_5,20_ = 3.59. Protein carbonyl blot density represented as mean ± SEM with individual data points, *N* = 4–8/group. (**B**) Cortical tissue sparing following mild brain contusion and subsequent pioglitazone administration. Mice received either sham surgery or CCI followed by pioglitazone (20 mg/kg/day) initiated at either 3 or 24 h post-injury. An osmotic pump was inserted to ensure delivery of 20 mg/kg/day; brains were then removed at 15 days post-injury, fixed, and prepared for cortical tissue sparing analysis. There was a significant decrease in cortical sparing following CCI as compared to sham groups. Pioglitazone treatment initiated at either 3 or 24 h after CCI was not significantly different compared to respective vehicle-treated CCI groups. One-way ANOVA, Tukey’s *post hoc*. **P < 0.01, *** *P* < 0.005. *F*_3,36_ = 7.48 (3 h initiation), *F*_3,42_ = 21.31 (24 h initiation). Per cent cortical sparing represented as mean ± SEM with individual data points, *N* = 6–20/group. Data were presented as percentage of contralateral cortical volume.

We do not see changes in protein carbonylation oxidative damage in total or synaptic mitochondrial fractions after mild CCI (Fig. 4A). Pioglitazone at 12 h administration had significantly lower PC as compared to vehicle CCI for glia-enriched mitochondria. PC activity among all groups was lower in total mitochondrial fractions compared to purified glia-enriched and synaptic fractions. In our efficacy study, we find that pioglitazone treatment initiated at either 3 or 24 h after mild brain contusion does not improve spared cortical tissue at 15 days post-injury (Fig. 4B).

## Discussion

Growing evidence suggests that mitochondrial function varies depending on specific cell types in the brain.^18^ Mitochondrial function in the synapse is critical as synaptic transmission and communication are heavily dependent upon mitochondrial regulation of ATP stores and calcium signalling.^19^ Dysfunction of presynaptic mitochondrial function disrupts synaptic homeostasis and contributes to the pathogenesis of neurodegenerative diseases, such as Alzheimer’s disease and Parkinson’s disease.^20,21^ Critically, synaptic mitochondria undergo high metabolic demand during injury or disease, which can propagate dysfunction.^11,22–24^ Based on our previous work,^11^ we now harness the capability of magnetic-activated cell sorting technology to isolate functional mitochondria from brain homogenate, which contains free-floating mitochondria, derived mainly from glia, as well as synaptosomes. Our refined workflow allows our group to assess mitochondrial function from distinct populations in various brain regions (Fig. 1).

This research builds upon our past studies examining pioglitazone efficacy in preclinical models of TBI.^4,8,10^ We now, for the first time, explore therapeutic potential of pioglitazone in the treatment of mild CCI or mild brain contusion. Our group has previously shown that graded severity of CCI results in graded levels of mitochondrial dysfunction, in which mild CCI produces relatively modest reductions in mitochondrial respiration.^25^ We directly demonstrate that total mitochondrial deficits from the injured cortex are due to dysfunction in synaptic mitochondria after CCI and immediate pioglitazone treatment can improve maximal mitochondrial bioenergetics after injury (Fig. 2). This corroborates our previous findings in a moderate CCI mouse model.^10^ However, we did find that delayed pioglitazone treatment did not improve mitochondrial function after mild brain contusion, which differs from the data in the model of severe CCI, as demonstrated in Hubbard *et al*.^4^

Given the limited depth of the CCI impact in this study, this mild contusion injury does not create a hippocampal lesion^26^; hence, we observe a lack of changes in mitochondrial respiration from total hippocampal mitochondria (Fig. 3). In distal regions to the impact site (penumbra), assessment of total mitochondria may not be sensitive enough to observe overt mitochondrial dysfunction. We show that refined synaptic samples may reveal further insight into therapeutic improvements (Fig. 3).

Protein carbonylation is protein oxidation that can be promoted by reactive oxygen species, which is a cascade of post-injury mitochondrial dysfunction. Indeed, several reports demonstrate increases following severe level of CCI.^4,13,27,28^ However, we do not see overt changes in PC oxidative damage in total or synaptic mitochondrial fractions after mild CCI (Fig. 4A). There is a reduction in PC in glia-enriched mitochondria when pioglitazone is administered at 12 h post-injury, although there were no bioenergetic deficits in this subpopulation. This corroborates our previous findings that deficits in mitochondrial bioenergetics can be present without overt changes in oxidative damage.^4^ Interestingly, PC levels among all groups are higher in glia-enriched mitochondria compared to synaptic fractions, showing possible cellular compartmentalization of PC.

Based on improvements with early (0.25 or 3 h) pioglitazone administration on mitochondrial function, we then conducted a study to compare 3 and 24 h treatment initiation after mild CCI. Pioglitazone treatment initiated at either 3 or 24 h after mild brain contusion does not improve spared cortical tissue (Fig. 4B). However, we found that pioglitazone administration provided modest cognitive benefits following mild CCI in the Supplementary data of our earlier published work.^4^ Further investigation is needed into improvements into network connectivity in the brain, neuronal function, and/or vascular health that pioglitazone provides after mild brain contusion. While pioglitazone does not spare overt brain tissue at 15 days post-injury, there may be various other pathological outcomes that are modulated by pioglitazone therapy.

We acknowledge several limitations of this study. This study only utilized male mice in the experimental design. Given the vast importance of incorporating both sexes and examining sex as a biological variable,^29,30^ our future steps will be to incorporate females. While this study does assay treatment in a mild model of CCI, this is still an open skull model. Therefore, incorporating closed skull models^15^ is of interest in our future investigation.

In our past research, we have demonstrated shifts in dose–response in subpopulations of mitochondria following spinal cord injury.^31^ We now demonstrate the direct advantage of refining mitochondrial populations to examine mitochondrial bioenergetics in models of disease or trauma. We again show that pioglitazone, a mitoNEET ligand, can restore mitochondrial dysfunction following mild brain contusion. Synaptic mitochondrial changes are relatively prevalent in the brain following mild focal brain contusion and can provide greater sensitivity to assess bioenergetic changes with injury and treatment. The culmination of this work paired with our previous study in a model of severe brain contusion^4^ highlights the need to examine therapies in varying TBI severities for a greater understanding of treatment efficacy.

## Supplementary Material

fcad032_Supplementary_Data

## Data Availability

The derived data that support the findings of this study are available from the corresponding author, upon reasonable request.

## Abbreviations

ANOVA =: analysis of variance
CCI =: controlled cortical impact
OCRs =: oxygen consumption rates
PC =: protein carbonyls
TBI =: traumatic brain injury
