# Supplementary material for: Pioglitazone restores mitochondrial function but does not spare cortical tissue following mild brain contusion
Source: Brain Commun. 2023 Feb 13;5(2):fcad032. doi: 10.1093/braincomms/fcad032 (PMC9985333; doi:10.1093/braincomms/fcad032)
Supplement: fcad032_Supplementary_Data [file fcad032_supplementary_data.zip › Original Submission.pdf]

OXFORD UNIVERSITY PRESS | Brain Communications

Pioglitazone restores mitochondrial function but does not spare cortical tissue following mild brain contusion

Journal:	*Brain Communications*
Manuscript ID	BRAINCOM-2022-317
Manuscript Type:	Original Article
Date Submitted by the Author:	08-Aug-2022
Complete List of Authors:	Hubbard, Brad; University of Kentucky, Spinal Cord and Brain Injury Research Center; University of Kentucky<br>Vekaria, Hemendra; University of Kentucky College of Medicine<br>Kalimon, Olivia J.; University of Kentucky; University of Kentucky, Department of Neuroscience<br>Spry, Malinda; University of Kentucky, Spinal Cord and Brain Injury Research Center<br>Brown, Emily P.; University of Kentucky<br>Kilbaugh, Todd J.; University of Pennsylvania, Department of Anesthesiology and Critical Care Medicine<br>Sullivan, Patrick; University of Kentucky College of Medicine, Neuroscience
Keywords:	Pioglitazone, mitochondrial, tbi, traumatic brain injury, mitochondria

SCHOLARONE™
Manuscripts

Pioglitazone restores mitochondrial function but does not spare cortical tissue following mild brain contusion

W. Brad Hubbard[1,2,3], Hemendra J. Vekaria[3], Olivia J. Kalimon[3,4], Malinda L. Spry[3], Emily P. Brown[3], Todd J. Kilbaugh[5], Patrick G. Sullivan[1,3,4]*

1. Lexington Veterans' Affairs Healthcare System
2. Department of Physiology, University of Kentucky
3. Spinal Cord and Brain Injury Research Center, University of Kentucky
4. Department of Neuroscience, University of Kentucky
5. Department of Anesthesiology and Critical Care Medicine, Children's Hospital of Philadelphia, Perelman School of Medicine, University of Pennsylvania, Philadelphia, Pennsylvania.

*denotes corresponding author

For Review Only

ABSTRACT

Pioglitazone was recently found to interact through the protein mitoNEET to improve mitochondrial function following traumatic brain injury (TBI). To provide broader evidence regarding the therapeutic effects of pioglitazone after TBI, the current study is focused on delayed therapy in a model of mild brain contusion. To assess pioglitazone therapy on brain bioenergetics, we use a technique to isolate sub-populations of total, glia-enriched, and synaptic mitochondria. Pioglitazone treatment was initially administered at 15min, 3h, 12h, and 24h following mild controlled cortical impact (CCI). At 48h post-injury, ipsilateral cortex and hippocampus were dissected and mitochondrial fractions were isolated. Maximal mitochondrial respiration injury-induced deficits were observed in total and synaptic fractions and 15min pioglitazone treatment following mild CCI was able to restore respiration to sham levels. While there are no injury-induced deficits in hippocampal fractions, we do find that 3h pioglitazone treatment after mild CCI can significantly increase maximal mitochondrial bioenergetics compared to vehicle-treated mild CCI group. However, delayed pioglitazone treatment initiated at either 3h or 24h after mild brain contusion does not improve spared cortical tissue. We demonstrate that synaptic mitochondrial deficits following mild focal brain contusion can be restored with early initiation of pioglitazone treatment. Further investigation is needed to determine functional improvements with pioglitazone beyond that of overt cortical tissue sparing following mild contusion TBI.

INTRODUCTION

Traumatic brain injury (TBI) continues to affect numerous individuals in the U.S. who suffer from the on-going consequences of post-TBI symptomatology [1]. As such, there are no FDA-approved therapeutics for TBI to alleviate on-going neurological disorders. An important hallmark and target of TBI pathophysiology is mitochondrial dysfunction. Mitochondria have numerous cellular functions, including ATP production, calcium buffering, and maintaining oxidant homeostasis. Importantly, protein signaling within mitochondria can regulate function and respiration. One such protein, mitoNEET, has been identified as an outer mitochondria membrane protein that can tightly regulate respiratory capacity, operating as a "power switch" of mitochondrial function [2, 3]. Importantly, ligands to mitoNEET, including pioglitazone, have proven efficacious in models of neurotrauma [4-10]. Even with the published evidence, no study to date has assessed pioglitazone treatment as mitochondrial-direct therapy in a mild severity model of brain contusion.

Our group have streamlined techniques to examine profiles of mitochondrial respiration from synaptic and non-synaptic (or glia-enriched) fractions [11]. In this study, we perform side-by-side comparison of total, glia-enriched, and synaptic respirometry from unique brain regions following traumatic brain injury (TBI). This short communication serves as a follow-up study to a Brain publication from our group [4]. This study was performed to increase efforts toward rigor and reproducibility in neurotrauma research by examining therapeutic efficacy in a mild model of focal brain contusion injury. We used a mild controlled cortical impact to assess pioglitazone therapy on mitochondrial subpopulation bioenergetic analysis and neuroprotection.

For Review Only

METHODS

Experimental Design

All of the studies performed were approved by the University of Kentucky Institutional Animal Care and Use Committee (IACUC), in compliance with the guidelines of the Association for the Assessment and Accreditation for Laboratory Animal Care, International and the NIH Guide for the Care and Use of Laboratory Animals [12]. Animal experiments complied with ARRIVE (Animal Research: Reporting of In Vivo Experiments) guidelines. All experiments were conducted using male C57BL/6J mice (2-3 months old; Jackson Laboratories, Bar Harbor, ME).

The animals were housed 5 per cage, maintained in a 14h light/10h dark cycle, fed a balanced diet ad libitum. Animals were randomly assigned to groups, based on injury designation (sham or CCI) and treatment designation (vehicle or pioglitazone). Treatments were given in random order. All experimentation was performed blinded to treatment groups. For all outcomes, experiments were conducted with biological replicates of n=4-8/group. Additionally, technical triplets were used in each assay.

Mice received a bolus (i.p.) administration of pioglitazone (100 µL volume; 1:1 DMSO and PEG400) at either 15 mins, 3hr, 12hr, 18hr or 24hr after mild CCI, based on the treatment strategy outlined in Hubbard, et al. [4] Experimental groups were euthanized at 48 h after injury or sham

procedures. Total, glia-enriched and synaptic mitochondrial bioenergetic measures were the endpoints for this study.

Controlled Cortical Impact

The CCI procedure was performed according to past studies with some modifications [4, 13]. Importantly, our model was modified to a mild CCI, incorporating cortical depression depth of 0.5mm with confined tissue loss to the cortical region[14]. Briefly, anesthetized (2.5% isoflurane) mice were fixed with ear bars in a stereotaxic frame. After scalp incision, a 3 mm craniotomy was performed lateral to midline and mice received a pneumatic impact (TBI-0310 Impactor, Precision Systems and Instrumentation, Fairfax, VA, USA: 0.5mm depth; 3.5m/sec velocity; 500msec dwell) directly to brain using a 2 mm impactor tip. Sham animals received a craniotomy but no impact. Following impact, the craniotomy was covered with absorbable hemostat (Surgicel). Mice recovered on a heating pad until the animals were fully responsive.

Total mitochondrial isolation

Mitochondria were isolated using previously employed differential mitochondrial isolation methods [15]. Animals were asphyxiated with CO_2 and rapidly decapitated. Following decapitation, the brain was rapidly removed, rinsed in ice-cold mitochondrial isolation buffer (IB), (215 mM Mannitol, 75 mM Sucrose, 0.1% BSA, 1 mM EGTA, and 20 mM HEPES at pH 7.2), and dissected on ice. Brain tissue samples from ipsilateral cortex (4mm tissue punch around lesion site), and ipsilateral hippocampus were homogenized using 8-10 strokes in Dounce homogenizers; 0.5ml of the 2ml tissue homogenate was placed in a 2ml microcentrifuge tube and then centrifuged at 1300 × G for 3 min. Centrifugation steps were performed as previously outlined [13, 15] and the resuspension was placed in a nitrogen cell disruptor (Parr Instrument Company, Moline, IL) at 1,200 psi for 10 min followed by rapid release of the pressure to release trapped synaptosomal mitochondria [16]. The pellets were resuspended in isolation buffer (without EGTA) to bring the approximate protein concentration to 10mg/ml. Protein concentration was determined using BCA protein assay kit (Pierce, Cat # 23,227) recording the absorbance at 560nm on Biotek Synergy HT plate reader (Winooski, VT).

Glia-enriched and synaptic mitochondrial isolation

For a detailed breakdown of the fractionated mitochondrial magnetic separation (FMMS) technique, see Hubbard, et al. 2019 [11]. Briefly, the remaining 1.5ml of homogenate (described above) from ipsilateral cortical tissue and ipsilateral hippocampus was taken and combined supernatants from the first and second low-speed spins were incubated for 30 min with anti-Tom22 microbeads (Miltenyi Biotec) at a concentration of 4 μL/1 mg wet tissue weight. The mixture was then added to MACS separation LS Columns, attached to Quadro MACS Separator (Miltenyi Biotec, cat. no. 130-097-040), to capture free mitochondria (glia-enriched fraction). The eluate was collected for subsequent use. The columns were plunged causing magnetically attached non-synaptic mitochondria to dissociate and enter into the 15ml conical tube. The glia-enriched

For Review Only

samples were centrifuged and protein was analyzed. The eluate sample was centrifuged to collect synaptosomes and the synaptic mitochondria was released using pressurized nitrogen cell disruptor. The resulting solution was incubated with anti-Tom22 microbeads (1 µL/1 mg wet tissue) for 30 min. All sequential steps were performed as previously described.

Measurement of Mitochondrial Bioenergetics

Mitochondrial bioenergetics were assayed in isolated mitochondria according to previous studies [4, 11]. Briefly, Seahorse XFe96 Flux Analyzer (Agilent Technologies, Santa Clara, CA) was used to measure oxygen consumption rates (OCRs) in the presence of mitochondrial substrates, inhibitors and uncouplers. On the day before the assay, the sensor cartridge of Extracellular Flux kit was hydrated according to the manufacturer's instructions. The day of the assay injection ports A to D of the sensor cartridge were loaded separately or in combinations of substrates/inhibitors/uncouplers to measure different states of respiration. Before loading, the stocks were diluted appropriately in respiration buffer (RB) (125mM KCl, 0.1% BSA, 20 mM HEPES, 2 mM MgCl2, and 2.5 mM KH2PO4, adjusted pH 7.2) such that after each sequential injection the final concentration of the modulators were 5 mM pyruvate, 2.5 mM malate and 1 mM ADP (via Port A), 2.5 µM oligomycin A (via Port B), 4 µM FCCP (via Port C) and 1 µM rotenone and 10mM of succinate (via Port D). 6µg total mitochondria, 3µg non-synaptic and 6µg synaptic mitochondrial protein were loaded per well in a volume of 30µl. The assay plates were centrifuged at 3,000 rpm for 4 min at 4°C. Additional respiration buffer was added to bring the starting volume to 175 µl for the XFe96. After calibration step, the utility plate was replaced by the assay plate carrying mitochondria. The assays were carried out under previously optimized conditions. Oxygen consumption rates (OCR) were measured for states of mitochondrial respiration. State III_{C1} response in presence of 5 mM pyruvate, 2.5 mM malate and 1 mM ADP (Port A) was measured followed by State IV response in presence of 2.5 µM oligomycin A (Port B). Sequentially, State V_{C1} and State V_{C2} OCR rates were recorded automatically in presence of 4 µM FCCP (Port C); 0.1 µM rotenone and 10 mM of succinate (Port D) respectively. Due to multiple cohorts utilized, OCR values were analyzed as percent sham values.

For Review Only

Protein carbonyl quantification

Mitochondrial homogenate aliquots (unused during respiration assays) were stored at -20°C until utilization for oxidative stress slot blots. Protein carbonyls (PC) were assessed in these samples as previously described [15]. Protein concentrations were determined using a BCA protein assay. Polyclonal RbxDNP (from Oxy Blot Protein Oxidation Kit, Chemicon-Millipore, Billerica, MA, Dilution 1:200; Cat No. S7150) was used for immunodetection. The membranes were scanned with a photo scanner (Epson Perfection V600, Long Beach, CA), and slot-blot line densities were quantified by the ImageQuant TL software package (GE Healthcare Bio-Sciences, Piscataway, NJ).

Brain Histology

One cohort of mice was euthanized at 15d post-injury by intraperitoneal injection of Fatal Plus (Vortech Pharmaceuticals, Dearborn, MI) before transcardial perfusion with cold, sterile saline followed by either cold 4% PFA. After perfusion, mice were decapitated and the brains were then removed from the skull and post-fixed in 4% PFA for 24h. Following post-fixation, tissue was placed into 1:1 4% PFA/30% sucrose mixture followed by 30% sucrose PBS buffer solution for at least 48h for cryoprotection. The brain tissue was cut into 35 µm thick coronal sections using a freezing stage, sliding microtome. A series of coronal tissue sections spaced ~400 µm apart were mounted on slides, stained with cresyl violet and subjected to image analysis for assessment of cortical tissue sparing. Quantitative assessment of cortical damage employed a blinded unbiased tracing protocol to compare ipsilateral cortex to contralateral cortex. All slides were assessed blindly with respect to treatment group, for ROI analysis to measure cortical sparing, using HALO quantitative tissue analysis software (Indica Labs; Albuquerque, NM). Data were converted to percentage of the contralateral cortex, which served as an internal control for each animal.

For Review Only

Statistics

Power analysis was conducted for experimental data a priori based on effect size and expected data variance. There were no exclusion criteria for this study. Statistical analysis was performed using Graph Pad Prism 8 (GraphPad Software, CA, USA). For all analyses, the significance of differences was set at $p < 0.05$. For mitochondrial and oxidative assays, one-way ANOVA was performed followed by a Dunnett's post hoc test with groups compared to Injured Vehicle, when appropriate. For tissue sparing, one-way ANOVA was performed followed by a Tukey's post hoc test.

RESULTS AND DISCUSSION

Growing evidence suggests that mitochondrial function varies depending on specific cell types in the brain [17]. Mitochondrial function in the synapse is critical as synaptic transmission and communication are heavily dependent upon mitochondrial regulation of ATP stores and calcium signaling [18]. Dysfunction of presynaptic mitochondrial function disrupts synaptic homeostasis and contributes to the pathogenesis of neurodegenerative diseases, such as Alzheimer's disease and Parkinson's disease [19, 20]. Critically, synaptic mitochondria undergo high metabolic demand during injury or disease, which can propagate dysfunction [11, 21-23]. Based on our previous work [11], we now harness the capability of magnetic-activated cell sorting (MACS) technology to isolate functional mitochondria from brain homogenate, which contains free-floating mitochondria, derived mainly from glia, as well as synaptosomes. Our refined workflow allows our group to assess mitochondrial function from distinct populations in various brain regions (Figure 1).

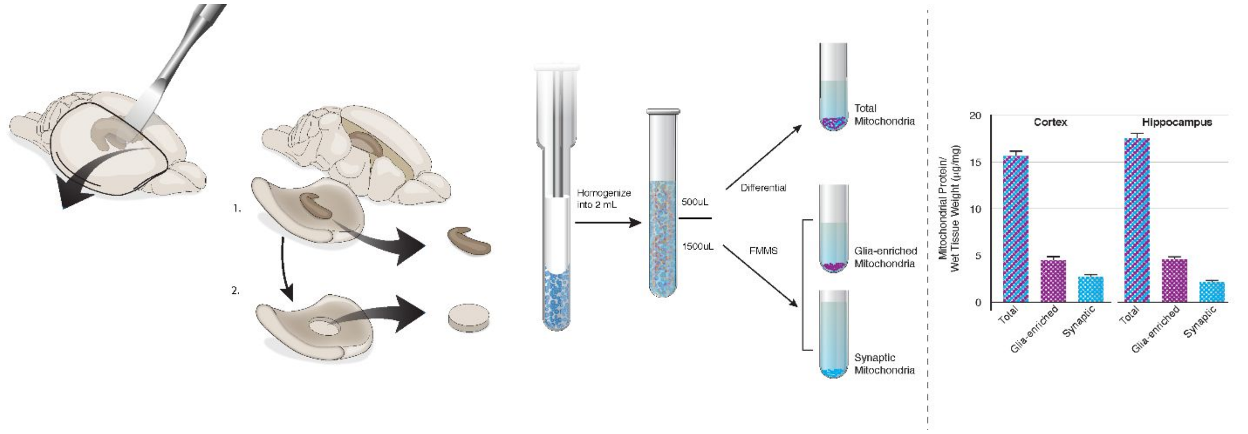

Figure 1. Schematic of the technical workflow and protein output. (Left) Dissection of ipsilateral hippocampus in (1) and cortical punch in (2) from a rodent model. (Middle) Homogenization of tissue resulting in fee-floating mitochondrial (glia-enriched) and well as synaptosomes. We utilized two protocols (differential and fractionated magnetic mitochondrial isolation) to derive three distinct mitochondrial sub-populations from the same sample. (Right) Mitochondrial protein output from three distinct populations from both the cortex and hippocampus. Protein was measured by BCA assay.

For Review Only

This research builds upon our past studies examining pioglitazone efficacy in preclinical models of TBI [4, 8, 10]. We now, for the first time, explore therapeutic potential of pioglitazone in the treatment of mild CCI or mild brain contusion. Our group has previously shown that graded severity of CCI results in graded levels of mitochondrial dysfunction, in which mild CCI produces relatively modest reductions in mitochondrial respiration [24]. We found that dysfunction in maximal mitochondrial respiration, mediated through both complex I and complex II, from cortical total mitochondria is aligned with deficits in synaptic mitochondrial function following mild CCI (Figure 2). Further, pioglitazone treatment at 15min post-injury can restore injury deficits in both total and synaptic populations of cortical mitochondria. There are no significant changes in mitochondrial respiration of glia-enriched cortical mitochondria following mild CCI. We directly demonstrate that total mitochondrial deficits from the injured cortex are due to dysfunction in synaptic mitochondria after CCI and immediate pioglitazone treatment can improve maximal mitochondrial bioenergetics after injury.

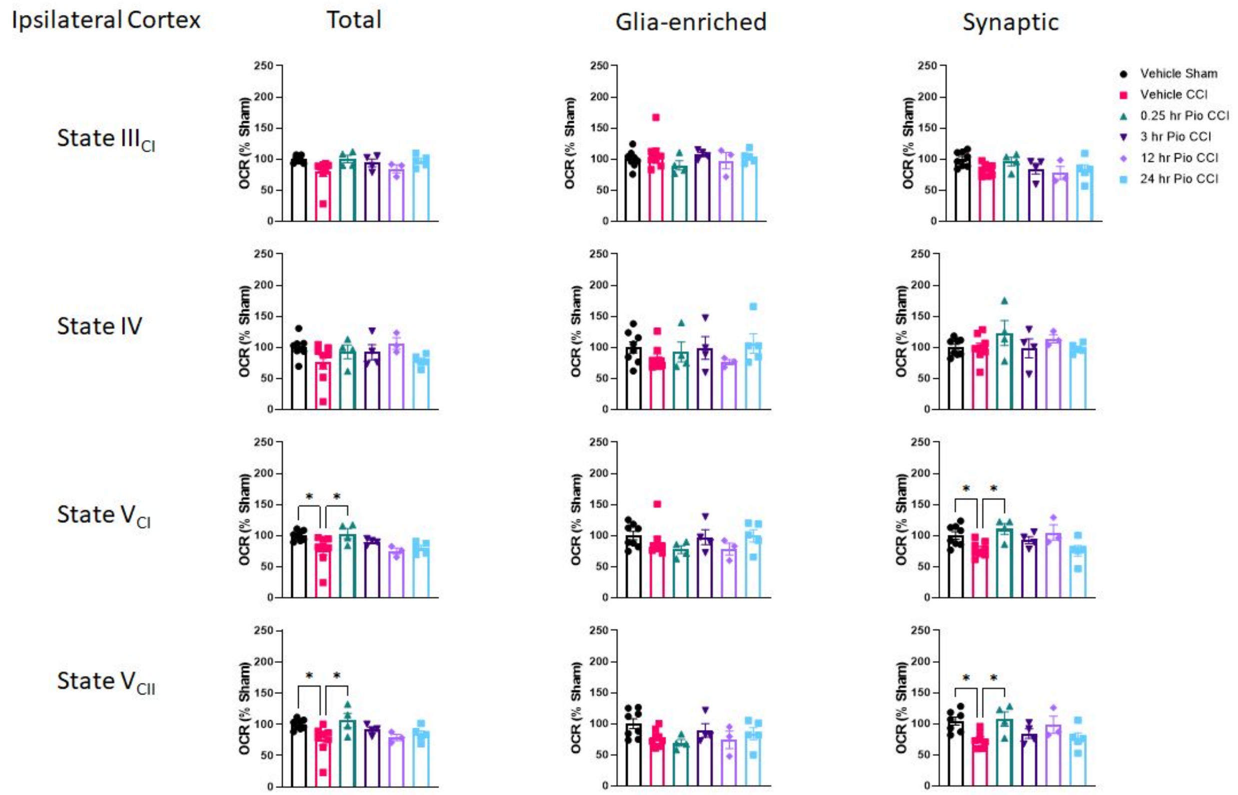

Figure 2. Mitochondrial bioenergetics from ipsilateral cortex following mild brain contusion and subsequent pioglitazone administration. Mice received either sham surgery or CCI followed by pioglitazone (20 mg/kg/day) initiated at either 0.25, 3, 12, or 24 hrs post-injury. An osmotic pump was inserted to ensure delivery of 20 mg/kg/day; mitochondria were then isolated from the ipsilateral cortex at 48hrs post-injury and bioenergetics were assayed using the Seahorse technology. There were no significant changes in State III$_{CI}$ or State IV mitochondrial bioenergetics between groups in any sub-population. However, there was a significant increase in State V$_{CI}$ respiration in Vehicle Sham and 0.25 hr Pio CCI groups compared to Vehicle CCI in total mitochondria. One-way ANOVA, Compared to Vehicle CCI, Dunnett's Post-Hoc. * $p<0.037$. $F_{5,26} = 3.525$. State V complex I-mediated maximal mitochondrial respiration was unaltered in glia-enriched mitochondria. In synaptic mitochondria, there was a significant increase in State V$_{CI}$ respiration in Vehicle Sham and 0.25 hr Pio CCI groups compared to Vehicle CCI. One-way ANOVA, Compared to Vehicle CCI, Dunnett's Post-Hoc. * $p<0.049$. $F_{5,26} = 4.14$. There was a significant increase in State V$_{CII}$ respiration in Vehicle Sham and 0.25 hr Pio CCI groups compared to Vehicle CCI in total mitochondria. One-way ANOVA, Compared to Vehicle CCI, Dunnett's Post-Hoc. * $p<0.022$. $F_{5,26} = 3.15$. State V complex II-mediated maximal mitochondrial respiration was unaltered in glia-enriched mitochondria. In synaptic mitochondria, there was a significant increase in State V$_{CII}$ respiration in Vehicle Sham and 0.25 hr Pio CCI groups compared to Vehicle CCI. One-way ANOVA, Compared to Vehicle CCI, Dunnett's Post-Hoc. * $p<0.029$. $F_{5,26} = 3.48$. Mean ± SEM with individual data points, N=4-8/group. Data presented as percentage of sham.

Given the limited depth of the CCI impact in this study, this mild contusion injury does not create a hippocampal lesion [25], hence we observe a lack of changes in mitochondrial respiration from total hippocampal mitochondria (Figure 3). In distal regions to the impact site (penumbra), assessment of total mitochondria may not be sensitive enough to observe overt mitochondrial dysfunction. There are also no significant changes in mitochondrial respiration of glia-enriched hippocampal mitochondria following mild CCI. However, we show delayed 3h pioglitazone-mediated increases in maximal mitochondrial respiration, mediated through both complex I and complex II, compared to vehicle CCI group in synaptic hippocampal mitochondria. We show that refined synaptic samples may reveal further insight into therapeutic improvements.

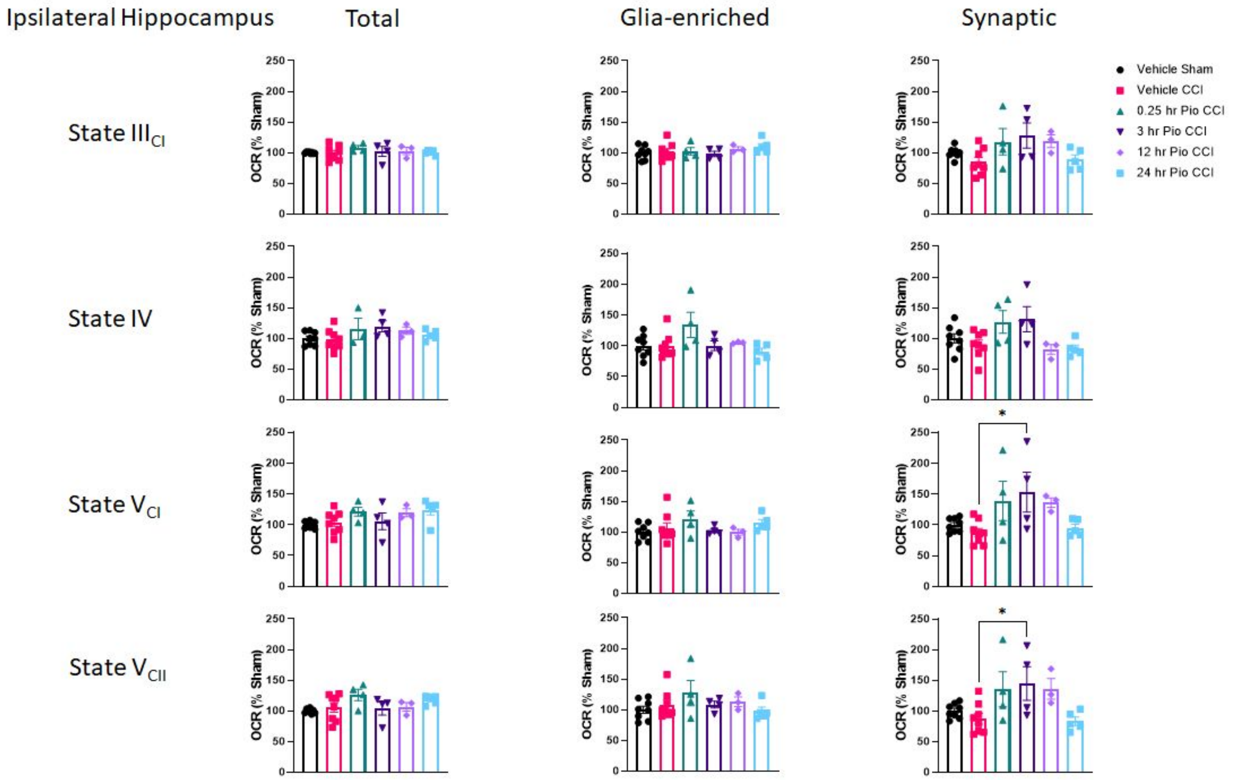

Figure 3. Mitochondrial bioenergetics from ipsilateral hippocampus following mild brain contusion and subsequent pioglitazone administration. Mice received either sham surgery or CCI followed by pioglitazone (20 mg/kg/day) initiated at either 0.25, 3, 12, or 24 hrs post-injury. An osmotic pump was inserted to ensure delivery of 20 mg/kg/day; mitochondria were then isolated from the ipsilateral hippocampus at 48hrs post-injury and bioenergetics were assayed using the Seahorse technology. There were no significant changes in State III$_{CI}$ or State IV mitochondrial bioenergetics between groups in any sub-population. There also were no changes in State V$_{CI}$ or State V$_{CII}$ respiration between groups for total and glia-enriched fractions. In synaptic mitochondria, there was a significant increase in State V$_{CI}$ respiration in 3 hr Pio CCI group compared to Vehicle CCI. One-way ANOVA, Compared to Vehicle CCI, Dunnett's Post-Hoc. *$p=0.017$. $F_{5,26} = 3.31$. There was also a significant increase in State V$_{CII}$ respiration in 3 hr Pio CCI group compared to Vehicle CCI in synaptic mitochondria. One-way ANOVA, Compared to

Vehicle CCI, Dunnett's Post-Hoc. * p=0.031. $F_{5,26} = 3.46$. Mean ± SEM with individual data points, N=4-8/group. Data presented as percentage of sham.

Protein carbonylation (PC) is protein oxidation that can be promoted by reactive oxygen species, which is a cascade of post-injury mitochondrial dysfunction. Indeed, several reports demonstrate increases following severe level of CCI [4, 13, 26, 27]. However, we do not see changes in PC oxidative damage in mitochondrial fractions after mild CCI (Figure 4A). It is possible that TBI-induced oxidative stress is observed in the cytosol, though we corroborate our previous findings that deficits in mitochondrial bioenergetics can be present without overt changes in oxidative damage [4]. PC activity among all groups was lower in total mitochondrial fractions compared to purified glia-enriched and synaptic fractions. Interestingly, protein carbonyl levels among all groups are higher in glia-enriched mitochondria compared to synaptic fractions, showing possible cellular compartmentalization of protein carbonyls.

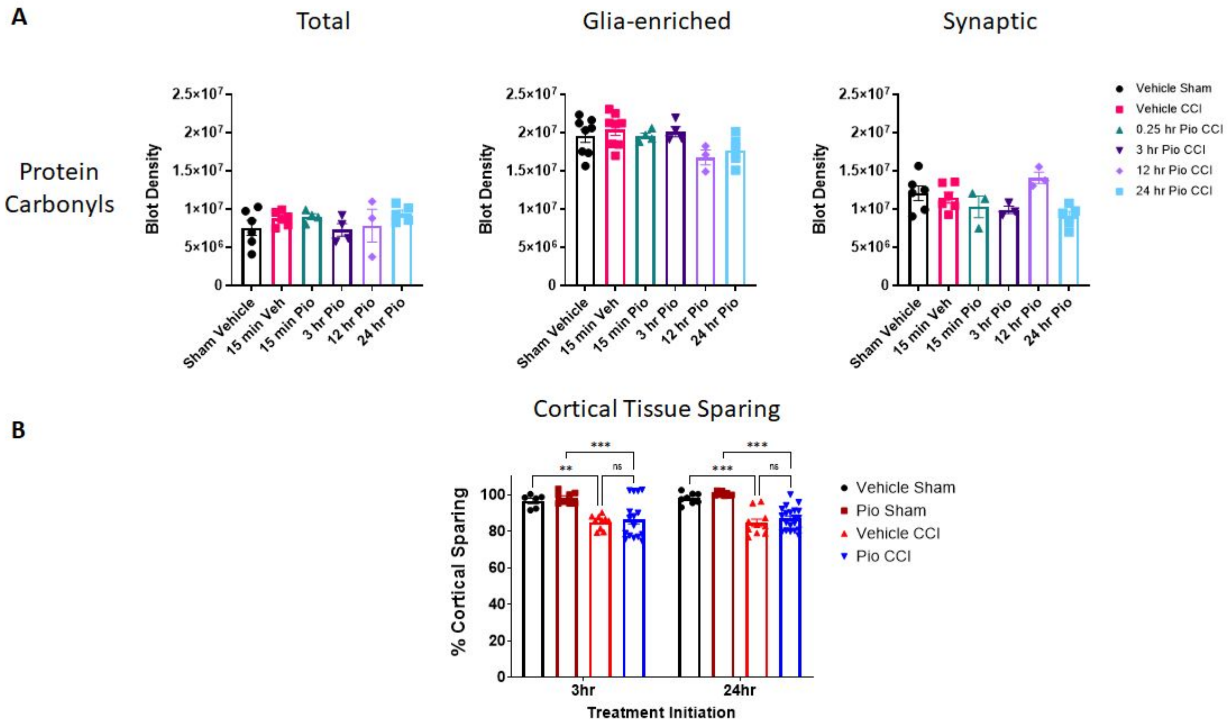

Figure 4. Pioglitazone does not alter oxidative markers or cortical tissue sparing. **A.** Protein carbonyl levels in the cortex following mild brain contusion and subsequent pioglitazone administration. There were no changes in protein carbonyl levels between groups for any mitochondrial sub-population. Mean ± SEM with individual data points, N=4-8/group. Data presented as percentage of sham. **B.** Cortical tissue sparing following mild brain contusion and subsequent pioglitazone administration. Mice received either sham surgery or CCI followed by pioglitazone (20 mg/kg/day) initiated at either 3 or 24 hrs post-injury. An osmotic pump was inserted to ensure delivery of 20 mg/kg/day; brains were then removed at 15d post-injury, fixed, and prepared for cortical tissue sparing analysis. There was a significant decrease in cortical

sparing following CCI as compared to sham groups. Pioglitazone treatment initiated at either 3h or 24h after CCI was not significantly different compared to respective vehicle-treated CCI groups. One-way ANOVA, Tukey's Post-Hoc. $^{**}p<0.01$, $^{***}p<0.005$. $F_{3,36} = 7.48$ (3h initiation), $F_{3,42} = 21.31$ (24h initiation). Mean ± SEM with individual data points, N=6-20/group. Data presented as percentage of contralateral cortical volume.

Based on improvements with early (15min or 3h) pioglitazone administration on mitochondrial function, we then conducted a study to compare 3h and 24h treatment initiation after mild CCI. In our efficacy study, we find that pioglitazone treatment initiated at either 3h or 24h after mild brain contusion does not improve spared cortical tissue (Figure 4B). However, we found that pioglitazone administration provided modest cognitive benefits following mild CCI in our earlier published work [4]. Further investigation is needed into improvements into network connectivity in the brain, neuronal function, and/or vascular health that pioglitazone provides after mild brain contusion. While pioglitazone does not spare overt brain tissue at 15d post-injury, there may be various other pathological outcomes it improves.

We acknowledge several limitations of this study. This study only utilized male mice in the experimental design. Given the vast importance of incorporating both sexes and examining sex as a biological variable [28, 29], our future steps will be to incorporate females. While this study does assay treatment in a mild model of CCI, this is still an open skull model. Therefore, incorporating closed skull models [15] is of interest in our future investigation.

In our past research, we have demonstrated shifts in dose-response in sub-populations of mitochondria following spinal cord injury [30]. We now demonstrate the direct advantage of refining mitochondrial populations to examine mitochondrial bioenergetics in models of disease or trauma. We again show that pioglitazone, a mitoNEET ligand, can restore mitochondrial dysfunction following mild brain contusion. Synaptic mitochondrial changes are relatively prevalent in the brain following mild focal brain contusion and can provide greater sensitivity to assess bioenergetic changes with injury and treatment. The culmination of our work [4] highlights the need to examine therapies in varying TBI severities for a greater understanding of treatment efficacy.

FUNDING

This work was supported by the Kentucky Spinal Cord and Head Injury Research Trust #20-7A (PGS). This work was supported in part by a Merit Review Award # I01BX003405-05A1 to (PGS) and Career Development Award Number IK2 BX004618 (WBH) from the United States Department of Veterans Affairs Biomedical Laboratory Research and Development Program. The contents do not represent the views of the U.S. Department of Veterans Affairs or the United States Government. This project was also supported by NIH R01 NS112693-01A1 (PGS).

REFERENCES

For Review Only

1. CDC. Report to Congress on Traumatic Brain Injury in the United States: Epidemiology and Rehabilitation. *National Center for Injury Prevention and Control; Division of Unintentional Injury Prevention*. 2015;Atlanta, GA
2. Wiley SE, Murphy AN, Ross SA, van der Geer P, Dixon JE. MitoNEET is an iron-containing outer mitochondrial membrane protein that regulates oxidative capacity. *Proceedings of the National Academy of Sciences*. 2007;104(13):5318-5323. doi:doi:10.1073/pnas.0701078104
3. Geldenhuys WJ, Leeper TC, Carroll RT. mitoNEET as a novel drug target for mitochondrial dysfunction. *Drug Discovery Today*. 2014/10/01/ 2014;19(10):1601-1606. doi:https://doi.org/10.1016/j.drudis.2014.05.001
4. Hubbard WB, Spry ML, Gooch JL, et al. Clinically relevant mitochondrial-targeted therapy improves chronic outcomes after traumatic brain injury. *Brain*. 2021;144(12):3788-3807. doi:10.1093/brain/awab341
5. McGuire JL, Correll EA, Lowery AC, et al. Pioglitazone improves working memory performance when administered in chronic TBI. *Neurobiol Dis*. Sep 9 2019;132:104611. doi:10.1016/j.nbd.2019.104611
6. Patel SP, Cox DH, Gollihue JL, et al. Pioglitazone treatment following spinal cord injury maintains acute mitochondrial integrity and increases chronic tissue sparing and functional recovery. *Experimental neurology*. Jul 2017;293:74-82. doi:10.1016/j.expneurol.2017.03.021
7. Rabchevsky AG, Patel SP, Sullivan PG. Targeting mitoNEET with pioglitazone for therapeutic neuroprotection after spinal cord injury. *Neural Regen Res*. Nov 2017;12(11):1807-1808. doi:10.4103/1673-5374.219040
8. Sauerbeck A, Gao J, Readnower R, et al. Pioglitazone attenuates mitochondrial dysfunction, cognitive impairment, cortical tissue loss, and inflammation following traumatic brain injury. *Exp Neurol*. Jan 2011;227(1):128-35. doi:10.1016/j.expneurol.2010.10.003
9. Semple BD, Noble-Haeusslein LJ. Broad-spectrum neuroprotection against traumatic brain injury by agonism of peroxisome proliferator-activated receptors. *Experimental neurology*. Jun 2011;229(2):195-7. doi:10.1016/j.expneurol.2011.02.002
10. Yonutas HM, Hubbard WB, Pandya JD, Vekaria HJ, Geldenhuys WJ, Sullivan PG. Bioenergetic restoration and neuroprotection after therapeutic targeting of mitoNEET: New mechanism of pioglitazone following traumatic brain injury. *Experimental Neurology*. 2020/05/01/ 2020;327:113243. doi:https://doi.org/10.1016/j.expneurol.2020.113243
11. Hubbard WB, Harwood CL, Prajapati P, Springer JE, Saatman KE, Sullivan PG. Fractionated mitochondrial magnetic separation for isolation of synaptic mitochondria from brain tissue. *Scientific Reports*. 2019/07/04 2019;9(1):9656. doi:10.1038/s41598-019-45568-3
12. National Research Council Committee for the Update of the Guide for the C, Use of Laboratory A. The National Academies Collection: Reports funded by National Institutes of Health. *Guide for the Care and Use of Laboratory Animals*. National Academies Press (US)

Copyright © 2011, National Academy of Sciences.; 2011.
13. Hubbard WB, Harwood CL, Geisler JG, Vekaria HJ, Sullivan PG. Mitochondrial uncoupling prodrug improves tissue sparing, cognitive outcome, and mitochondrial bioenergetics after traumatic brain injury in male mice. *J Neurosci Res*. Oct 2018;96(10):1677-1688. doi:10.1002/jnr.24271
14. Siebold L, Obenaus A, Goyal R. Criteria to define mild, moderate, and severe traumatic brain injury in the mouse controlled cortical impact model. *Experimental Neurology*. 2018/12/01/ 2018;310:48-57. doi:https://doi.org/10.1016/j.expneurol.2018.07.004
15. Hubbard WB, Joseph B, Spry M, Vekaria HJ, Saatman KE, Sullivan PG. Acute Mitochondrial Impairment Underlies Prolonged Cellular Dysfunction after Repeated Mild Traumatic Brain Injuries. *J Neurotrauma*. Apr 15 2019;36(8):1252-1263. doi:10.1089/neu.2018.5990

For Review Only

16. Brown MR, Sullivan PG, Dorenbos KA, Modafferi EA, Geddes JW, Steward O. Nitrogen disruption of synaptoneurosomes: an alternative method to isolate brain mitochondria. *J Neurosci Methods*. Aug 30 2004;137(2):299-303. doi:10.1016/j.jneumeth.2004.02.028
17. Fecher C, Trovò L, Müller SA, et al. Cell-type-specific profiling of brain mitochondria reveals functional and molecular diversity. *Nature Neuroscience*. 2019/10/01 2019;22(10):1731-1742. doi:10.1038/s41593-019-0479-z
18. Jonas E. Regulation of synaptic transmission by mitochondrial ion channels. *J Bioenerg Biomembr*. Aug 2004;36(4):357-61. doi:10.1023/b:Jobb.0000041768.11006.90
19. Devine MJ, Kittler JT. Mitochondria at the neuronal presynapse in health and disease. *Nature Reviews Neuroscience*. 2018/02/01 2018;19(2):63-80. doi:10.1038/nrn.2017.170
20. Lee A, Hirabayashi Y, Kwon S-K, Lewis TL, Polleux F. Emerging roles of mitochondria in synaptic transmission and neurodegeneration. *Current Opinion in Physiology*. 2018/06/01/ 2018;3:82-93. doi:https://doi.org/10.1016/j.cophys.2018.03.009
21. Ansari MA, Roberts KN, Scheff SW. A time course of contusion-induced oxidative stress and synaptic proteins in cortex in a rat model of TBI. *Journal of neurotrauma*. May 2008;25(5):513-26. doi:10.1089/neu.2007.0451
22. Hill RL, Kulbe JR, Singh IN, Wang JA, Hall ED. Synaptic Mitochondria are More Susceptible to Traumatic Brain Injury-induced Oxidative Damage and Respiratory Dysfunction than Non-synaptic Mitochondria. *Neuroscience*. Aug 21 2018;386:265-283. doi:10.1016/j.neuroscience.2018.06.028
23. Kulbe JR, Hill RL, Singh IN, Wang JA, Hall ED. Synaptic Mitochondria Sustain More Damage than Non-Synaptic Mitochondria after Traumatic Brain Injury and Are Protected by Cyclosporine A. *J Neurotrauma*. 2017;34(7):1291-1301. doi:10.1089/neu.2016.4628
24. Gilmer LK, Roberts KN, Joy K, Sullivan PG, Scheff SW. Early mitochondrial dysfunction after cortical contusion injury. *J Neurotrauma*. Aug 2009;26(8):1271-80. doi:10.1089/neu.2008.0857
25. Saatman KE, Feeko KJ, Pape RL, Raghupathi R. Differential behavioral and histopathological responses to graded cortical impact injury in mice. *J Neurotrauma*. Aug 2006;23(8):1241-53. doi:10.1089/neu.2006.23.1241
26. Hill RL, Singh IN, Wang JA, Hall ED. Time courses of post-injury mitochondrial oxidative damage and respiratory dysfunction and neuronal cytoskeletal degradation in a rat model of focal traumatic brain injury. *Neurochemistry international*. Dec 2017;111:45-56. doi:10.1016/j.neuint.2017.03.015
27. Singh IN, Sullivan PG, Deng Y, Mbye LH, Hall ED. Time course of post-traumatic mitochondrial oxidative damage and dysfunction in a mouse model of focal traumatic brain injury: implications for neuroprotective therapy. *J Cereb Blood Flow Metab*. Nov 2006;26(11):1407-18. doi:10.1038/sj.jcbfm.9600297
28. Gupte R, Brooks W, Vukas R, Pierce J, Harris J. Sex Differences in Traumatic Brain Injury: What We Know and What We Should Know. *Journal of neurotrauma*. Nov 15 2019;36(22):3063-3091. doi:10.1089/neu.2018.6171
29. Hubbard WB, Velmurugan GV, Brown EP, Sullivan PG. Resilience of females to acute blood-brain barrier damage and anxiety behavior following mild blast traumatic brain injury. *Acta Neuropathol Commun*. Jun 27 2022;10(1):93. doi:10.1186/s40478-022-01395-8
30. Patel SP, Sullivan PG, Pandya JD, et al. N-acetylcysteine amide preserves mitochondrial bioenergetics and improves functional recovery following spinal trauma. *Exp Neurol*. Jul 2014;257:95-105. doi:10.1016/j.expneurol.2014.04.026

For Review Only

The ARRIVE guidelines 2.0: author checklist

The ARRIVE Essential 10

These items are the basic minimum to include in a manuscript. Without this information, readers and reviewers cannot assess the reliability of the findings.

Item		Recommendation	Section/line number, or reason for not reporting
**Study design**	1	For each experiment, provide brief details of study design including:<br>a. The groups being compared, including control groups. If no control group has been used, the rationale should be stated.<br>b. The experimental unit (e.g. a single animal, litter, or cage of animals).	METHODS Experimental Design
**Sample size**	2	a. Specify the exact number of experimental units allocated to each group, and the total number in each experiment. Also indicate the total number of animals used.	Figures/Figure legends
		b. Explain how the sample size was decided. Provide details of any *a priori* sample size calculation, if done.	Methods; Statistics
**Inclusion and exclusion criteria**	3	a. Describe any criteria used for including and excluding animals (or experimental units) during the experiment, and data points during the analysis. Specify if these criteria were established *a priori*. If no criteria were set, state this explicitly.	Methods; Statistics
		b. For each experimental group, report any animals, experimental units or data points not included in the analysis and explain why. If there were no exclusions, state so.	
		c. For each analysis, report the exact value of *n* in each experimental group.	See Figure legends
**Randomisation**	4	a. State whether randomisation was used to allocate experimental units to control and treatment groups. If done, provide the method used to generate the randomisation sequence.<br>b. Describe the strategy used to minimise potential confounders such as the order of treatments and measurements, or animal/cage location. If confounders were not controlled, state this explicitly.	METHODS Experimental Design
**Blinding**	5	Describe who was aware of the group allocation at the different stages of the experiment (during the allocation, the conduct of the experiment, the outcome assessment, and the data analysis).	METHODS Experimental Design
**Outcome measures**	6	a. Clearly define all outcome measures assessed (e.g. cell death, molecular markers, or behavioural changes).<br>b. For hypothesis-testing studies, specify the primary outcome measure, i.e. the outcome measure that was used to determine the sample size.	see Methods
**Statistical methods**	7	a. Provide details of the statistical methods used for each analysis, including software used.<br>b. Describe any methods used to assess whether the data met the assumptions of the statistical approach, and what was done if the assumptions were not met.	Methods; Statistics
**Experimental animals**	8	a. Provide species-appropriate details of the animals used, including species, strain and substrain, sex, age or developmental stage, and, if relevant, weight.<br>b. Provide further relevant information on the provenance of animals, health/immune status, genetic modification status, genotype, and any previous procedures.	METHODS Experimental Design
**Experimental procedures**	9	For each experimental group, including controls, describe the procedures in enough detail to allow others to replicate them, including:<br>a. What was done, how it was done and what was used.<br>b. When and how often.<br>c. Where (including detail of any acclimatisation periods).<br>d. Why (provide rationale for procedures).	see Methods
**Results**	10	For each experiment conducted, including independent replications, report:<br>a. Summary/descriptive statistics for each experimental group, with a measure of variability where applicable (e.g. mean and SD, or median and range).<br>b. If applicable, the effect size with a confidence interval.	see Figures

For Review Only

University of Kentucky

Patrick G. Sullivan, Ph.D.
Professor of Neuroscience and Endowed Chair,
Spinal Cord & Brain Injury Research Center (SCoBIRC)
University of Kentucky College of Medicine
475 Biomedical & Biological Sciences Research Bldg
741 South Limestone St Lexington, KY 40536-0509
(859) 323-4684 – office
(859) 323-4682 – lab
(859) 257-5737 – fax
PatSullivan@uky.edu

August 3, 2022

Brain Communications
Editorial Board

Dear Editor in Chief:

We would like to submit our article entitled, **“Pioglitazone restores mitochondrial function but does not spare cortical tissue following mild brain contusion”**, for consideration and possible publication in *Brain Communications*. Importantly, this study directly ties into the recently published article in *Brain*, “Clinically relevant mitochondrial-targeted therapy improves chronic outcomes after traumatic brain injury.” This manuscript highlights a novel, validated technique to isolate total, glia-enriched, and synaptic mitochondria from brain tissue to assess injury mechanisms and therapeutic efficacy. We demonstrate a comparative analysis of mitochondrial function from these distinct mitochondrial subpopulations from different brain regions. We apply this technique to the assessment of pioglitazone therapy in our experimental traumatic brain injury (TBI) model. We show that synaptic populations are susceptible to injury and disease.

On behalf of all of the authors, I hereby declare that none of this material has been published or is under consideration at any other journal, including the Internet. All of the authors have reviewed this manuscript and agree to its contents. All animal studies were conducted using ARRIVE guidelines and approved by appropriate IACUC committees.

Thank you so much for your time and consideration. Please feel free to contact me with any questions or concerns.

Sincerely,

Patrick G. Sullivan, Ph.D.

For Review Only
