# Supplementary material for: Pioglitazone restores mitochondrial function but does not spare cortical tissue following mild brain contusion
Source: Brain Commun. 2023 Feb 13;5(2):fcad032. doi: 10.1093/braincomms/fcad032 (PMC9985333; doi:10.1093/braincomms/fcad032)
Supplement: fcad032_Supplementary_Data [file Original Submission - Author Response to Reviewers.pdf › Original Submission - Author Response to Reviewers.pdf]

Original Submission – Author Response to Reviewers

We thank both reviewers for the constructive comments as well as the time and effort in reviewing our manuscript. Please see our point-to-point responses (underlined) provided below:

Reviewer 1

The manuscript by Hubbard B. et al., studies the therapeutic effects of pioglitazone in a model of mild brain contusion injury by analyzing mitochondrial subpopulations bioenergetics. Potentially this is an interesting study, however some improvements should be done.

Specific points

Major 1.

Although the authors mentioned that this manuscript serves as a follow-up study to their previous publication, adding more details about the previous findings in the introduction/results paragraphs and about the techniques in the method section would be appropriate and would give a better overview to the readers.

We have now given more detail regarding the findings of our previous Brain publication (see second paragraph of the Introduction and throughout the Results section). The methods contain all techniques and details relevant to this current study.

2. It is not clear why the authors defined the manuscript as "short communication" since it has been submitted as "original article"; therefore, it would be appropriate to describe the results in a separate section from the discussion.

Thank you. We have now separated the Results section from the Discussion section.

3. The experimental design is not clear. It is not explained why some animals are sacrificed 48h post injury and some 15 days. In the methods different cohorts of animals are mentioned but not really described.

Thank you for the comment. We now describe this fully in Methods; Experimental Design.

4. Page 9 line 13: the authors mentioned the possibility of oxidative stress in the cytosol but did not show any data.

Thank you for pointing this out. We have removed this statement.

5. The use of only male animals is a limitation, as the authors already mentioned; moreover, expanding the analyses to the use of the contralateral hippocampus would represent a proper control.

Our studies utilize sham ipsilateral hippocampus as a control to account for possible secondary damage that could occur in the contralateral hemisphere of injured mice.

Minor

1.

It would be appropriate to be consistent when the authors refer to the pioglitazone administration times.

<u>Thank you for the comment and we have improved this consistency (i.e. 0.25hr).</u>

2. It would be appropriate to be consistent with the use of Roman or Arabic numbers when the authors refer to the State III/IV/V C1-1 (or CI-II) in the method “measurement of mitochondrial bioenergetics” and in the figures 2 and 3.

<u>Thank you and this is updated.</u>

3. Page 7, figure 2: for the first time the authors use the acronym Pio without specifying it before.

<u>We have know defined this acronym in the Figure legends.</u>

Reviewer 2

Summary:

This study evaluated treatment with pioglitazone after brain contusion in adult male mice. Pioglitazone binds to mitoNEET in the outer mitochondrial memberane, and has a growing body of evidence supporting its beneficial effects in various preclinical models of neurotrauma. However, it has not yet been tested in a model of mild TBI. The current study serves as a follow-up to a recent publication by this group, extending previous results by using a milder “dose” of brain trauma.

Findings indicate functional deficits in mitochondrial complex I and II in the injured cortex that are rescued by pioglitazone treatment. However, pioglitazone did not alter markers of oxidative status nor improve cortical tissue sparing.

This is a very nice follow-up to the authors’ recent work that will be suitable for publication in Brain Communications after the following critiques are addressed.

Comments and critiques:

Overall, the study appears to have been well executed, and the data from a rather complex study design are very clearly presented in the graphs. Limitations are clearly addressed. The writing is clear and concise. A few grammatical errors remain that should be fixed by a careful editor.

<u>We believe grammatical errors are now fixed.</u>

Additional information on the CCI would be helpful. Was the dura disrupted by the craniectomy in sham and/or CCI mice? Was the skull flap replaced? How long did the procedure take, and was the length of anesthesia the same for CCI and sham mice?

<u>Thank you. These details are now added in the Methods section. The skull flap was not replaced.</u>

For OCR values run as percent of sham, please specify if shams were run on the same plate, and how many. Also please clarify how you calculated sham data as percent of sham.

Thank you. This is now clarified in the section “Measurement of Mitochondrial Bioenergetics.”

An important concern is the characterization of this animal model as “mild TBI”. Despite the physical characteristics of the impact delivered, by 15 days the percent cortical damage appears to be close to 20% (i.e. 80% spared cortex) which is in a similar range to the amount of damage reported in your recent Brain paper (Figure 4) which characterized a “severe” contusive injury! This begs some additional explanation and discussion.

The authors were careful to state this model is mild severity CCI or mild brain contusion. However, we greatly appreciate your comment as this model is at a milder severity than that reported in the Brain paper. In examining our analysis, it appears that only half of each hemi-cortex was traced, which caused the data to appear as if there was twice the amount of lesion area percentage as there actually was. Our methodology was made consistent and the data now represents a contusion level consistent with mild CCI.

The description of the power analysis is insufficient. What type of data were used for the power analysis (analyses)? What was the effect size and variance in preliminary data used for power analysis?

Thank you for addressing this oversight. This has now been expanded upon.

Re “no exclusion criteria for this study” please clarify. Does this mean that all animals entered into the study were used? All Seahorse plates and replicates were used? Etc.

This is now clarified.

Figure 1. Suggest editing this figure for clarity, as the text is very hard to read.

The journal suggested including all figures directly in the Word document, which compromised figure resolution. The issue could be solved now that figures are submitted directly to the submission site.

Figure 2 and 3. It would be helpful to see the Y-axis expanded so individual values in some of those clusters of points can be better seen.

The y-axes are now expanded to the maximum amount to ensure comparability across respiration states as well as inclusion of all data points.

“…cognitive benefits following mild CCI in our earlier published work4.” The paper cited is not a mTBI model.

The data referenced was included in the Supplementary data of the previous manuscript and this is now clarified.

The experiments as described in the manuscript do not fully address all areas on the ARRIVE checklist. A closer review of the ARRIVE guidelines is encouraged.

Thank you and we worked with the Scientific Editor to improve our adherence.

Comments from the Scientific Editor:

For all the figures, please, report what each data point represents in the legend.

Thank you – this is completed.

Fig4a: please report in the figure legend the test used and relevant test statistic values alongside p values (for example F or t values for ANOVAs and t-tests).

Thank you – this is completed.

Please include the page numbers corresponding to the items listed in the reporting guideline checklist.

Thank you – this is completed.

Please supply a graphical abstract. A graphical abstract is a visual representation of the central finding or methodology of the paper. It will be displayed in the table of contents. Authors should strive to make the graphical abstract informative, interesting, visually appealing, and straightforward (no caption needed, no abbreviations except gene names). The technical requirements for the graphical abstracts are:

- Size: 120 x 120 mm (300-600dpi) or 3000 x 3000 pixels

- Font: Arial 12-16 pt

- File type: tiff or PDF

- Aspect ratio: 1:1 preferred

This is now supplied.

Please add an Abbreviated Summary (up to 50 words) of your paper that captures the main purpose and conclusions of the work. The summary should state the main results written in the third person ('[1st author surname] et al. report that …') and conclusion. This summary will be used in the contents list online and should not contain any abbreviations other than those on the accepted abbreviations list and accepted gene/protein names, making it accessible to the non-specialist. Please upload it as a Word document. Allowed abbreviations: AIDS; ANOVA; AMPA; ATP; A,T,C,G; CNS; CSF; CT; DNA; ECG; EEG; EMG; GABA; HIV; MRI; NMDA; PET; PCR; RNA.

This is now supplied.

Please include a paragraph about data availability in the 'Materials and Methods section. We strongly encourage data sharing either as supplementary information or uploads to repositories which you can link in the paper.

This is now included.
