# Supplementary material for: Pioglitazone restores mitochondrial function but does not spare cortical tissue following mild brain contusion
Source: Brain Commun. 2023 Feb 13;5(2):fcad032. doi: 10.1093/braincomms/fcad032 (PMC9985333; doi:10.1093/braincomms/fcad032)
Supplement: fcad032_Supplementary_Data [file Original submission - Decision Letter.pdf › Original submission - Decision Letter.pdf]

Original Submission – Decision letter

22-Sep-2022

Dear Dr. Sullivan,

Thank you for your recent submission BRAINCOM-2022-317 entitled "Pioglitazone restores mitochondrial function but does not spare cortical tissue following mild brain contusion" to Brain Communications.

Your manuscript has been reviewed, and while the reviewers and editorial team appreciate many aspects of the paper, they raise some concerns that you will need to address in order for the paper to be considered for publication.

If you can address these concerns, I invite you to submit a revised manuscript and a response to each of the reviewers' comments. The reviewer(s)' comments are included at the bottom of this message.

In addition to the reviewers' comments, please also address our Scientific Editor's comments added below to comply with our requirements for presentation of scientific data at Brain Communications.

Please format your revised manuscript according to our Instructions to Authors https://academic.oup.com/braincomms/pages/General_Instructions – a summary of key points is attached to help with this.

To revise your manuscript, click the link below or log into https://mc.manuscriptcentral.com/braincom and enter your Author Centre, where you will find your manuscript title listed under "Manuscripts with Decisions." Under "Actions," click on "Create a Revision." Your manuscript number will be appended to denote a revision.

Please upload a revised manuscript with changes tracked with track changes or in coloured text.

When submitting your revised manuscript, you will be asked to respond to the reviewer(s) comments. Please be as specific as possible in your response to all of the comments.

The editorial team at Brain Communications aims to increase transparency in scientific publishing and to demonstrate the value that peer review brings to the scientific process. Please therefore consider whether you are willing to have reviewer(s)' comments on your paper and your response published as supplementary data with the paper if it is accepted. This is entirely optional and will only be done if both the authors and reviewers agree. While submitting your revision there will be an opportunity to review or update the answer you gave previously, if necessary.

IMPORTANT: Your original files are available to you when you upload your revised manuscript. Please delete any redundant files before completing the submission.

Because we are trying to facilitate timely publication of manuscripts submitted to Brain Communications, we would appreciate your revised manuscript as soon as possible. If it is not possible for you to resubmit within 3 months, the option to submit a revision will expire, and we would then consider your revised paper as a new submission. However, if you need a little more time, please get in touch and we can extend this deadline for you.

Once again, thank you for submitting your manuscript to Brain Communications and I look forward to receiving your revision.

Best wishes

Dr. Rakez Kayed

Associate Editor, Brain Communications

Professor Tara Spires-Jones
Founding Editor, Brain Communications

Reviewer(s)' Comments to Author:

Reviewer: 1 (Michela Marcatti)

Comments to the Author
The manuscript is interesting although some improvements would be needed before publication.

The manuscript by Hubbard B. et al., studies the therapeutic effects of pioglitazone in a model of mild brain contusion injury by analyzing mitochondrial subpopulations bioenergetics. Potentially this is an interesting study, however some improvements should be done.

Specific points

Major

1. Although the authors mentioned that this manuscript serves as a follow-up study to their previous publication, adding more details about the previous findings in the introduction/results paragraphs and about the techniques in the method section would be appropriate and would give a better overview to the readers.
2. It is not clear why the authors defined the manuscript as "short communication" since it has been submitted as "original article"; therefore, it would be appropriate to describe the results in a separate section from the discussion.
3. The experimental design is not clear. It is not explained why some animals are sacrificed 48h post injury and some 15 days. In the methods different cohorts of animals are mentioned but not really described.
4. Page 9 line 13: the authors mentioned the possibility of oxidative stress in the cytosol but did not show any data.
5. The use of only male animals is a limitation, as the authors already mentioned; moreover, expanding the analyses to the use of the contralateral hippocampus would represent a proper control.

Minor

1. It would be appropriate to be consistent when the authors refer to the pioglitazone administration times.
2. It would be appropriate to be consistent with the use of Roman or Arabic numbers when the authors refer to the State III/IV/V C1-1 (or CI-II) in the method "measurement of mitochondrial bioenergetics" and in the figures 2 and 3.
3. Page 7, figure 2: for the first time the authors use the acronym Pio without specifying it before.

Reviewer: 2 (Janna Harris)

Comments to the Author
Summary:
This study evaluated treatment with pioglitazone after brain contusion in adult male mice. Pioglitazone binds to mitoNEET in the outer mitochondrial memberane, and has a growing body of evidence supporting its beneficial effects in various preclinical models of neurotrauma. However, it has not yet been tested in a model of mild TBI. The current study serves as a follow-up to a recent publication by this group, extending previous results by using a milder "dose" of brain trauma.

Findings indicate functional deficits in mitochondrial complex I and II in the injured cortex that are rescued by pioglitazone treatment. However, pioglitazone did not alter markers of oxidative status nor improve cortical tissue sparing.

This is a very nice follow-up to the authors' recent work that will be suitable for publication in Brain Communications after the following critiques are addressed.

Comments and critiques:

Overall, the study appears to have been well executed, and the data from a rather complex study design are very clearly presented in the graphs. Limitations are clearly addressed. The writing is clear and concise. A few grammatical errors remain that should be fixed by a careful editor.

Additional information on the CCI would be helpful. Was the dura disrupted by the craniectomy in sham and/or CCI mice? Was the skull flap replaced? How long did the procedure take, and was the length of anesthesia the same for CCI and sham mice?

For OCR values run as percent of sham, please specify if shams were run on the same plate, and how many. Also please clarify how you calculated sham data as percent of sham.

An important concern is the characterization of this animal model as "mild TBI". Despite the physical characteristics of the impact delivered, by 15 days the percent cortical damage apears to be close to 20% (i.e. 80% spared cortex) which is in a similar range to the amount of damage reported in your recent Brain paper (Figure 4) which characterized a "severe" contusive injury! This begs some additional explanation and discussion.

The description of the power analysis is insufficient. What type of data were used for the power analysis (analyses)? What was the effect size and variance in preliminary data used for power analysis?

Re "no exclusion criteria for this study" please clarify. Does this mean that all animals entered into the study were used? All Seahorse plates and replicates were used? Etc.

Figure 1. Suggest editing this figure for clarity, as the text is very hard to read.

Figure 2 and 3. It would be helpful to see the Y-axis expanded so individual values in some of those clusters of points can be better seen.

"…cognitive benefits following mild CCI in our earlier published work4." The paper cited is not a mTBI model.

The experiments as described in the manuscript do not fully address all areas on the ARRIVE checklist. A closer review of the ARRIVE guidelines is encouraged.

Comments from the Scientific Editor:

For all the figures, please, report what each data point represents in the legend.

Fig4a: please report in the figure legend the test used and relevant test statistic values alongside p values (for example F or t values for ANOVAs and t-tests).

Please include the page numbers corresponding to the items listed in the reporting guideline checklist.

Please supply a graphical abstract. A graphical abstract is a visual representation of the central finding or methodology of the paper. It will be displayed in the table of contents. Authors should strive to make the graphical abstract informative, interesting, visually appealing, and straightforward (no caption needed, no abbreviations except gene names). The technical requirements

for the graphical abstracts are:
- Size: 120 x 120 mm (300-600dpi) or 3000 x 3000 pixels
- Font: Arial 12-16 pt
- File type: tiff or PDF
- Aspect ratio: 1:1 preferred

Please add an Abbreviated Summary (up to 50 words) of your paper that captures the main purpose and conclusions of the work. The summary should state the main results written in the third person (‘[1st author surname] et al. report that …’) and conclusion. This summary will be used in the contents list online and should not contain any abbreviations other than those on the accepted abbreviations list and accepted gene/protein names, making it accessible to the non-specialist. Please upload it as a Word document. Allowed abbreviations: AIDS; ANOVA; AMPA; ATP; A,T,C,G; CNS; CSF; CT; DNA; ECG; EEG; EMG; GABA; HIV; MRI; NMDA; PET; PCR; RNA.

Please include a paragraph about data availability in the 'Materials and Methods' section. We strongly encourage data sharing either as supplementary information or uploads to repositories which you can link in the paper.
