# Supplementary material for: Pioglitazone restores mitochondrial function but does not spare cortical tissue following mild brain contusion
Source: Brain Commun. 2023 Feb 13;5(2):fcad032. doi: 10.1093/braincomms/fcad032 (PMC9985333; doi:10.1093/braincomms/fcad032)
Supplement: fcad032_Supplementary_Data [file Revision 1 - Author Response to Reviewers.pdf › Revision 1 - Author Response to Reviewers.pdf]

Revision 1 – Author Response to Reviewers

We thank both reviewers for their positive comments in addition to the time and effort that goes into manuscript review.

We have replaced and rephrased portions of our Materials and Methods (including Total mitochondrial isolation, Measurement of mitochondrial bioenergetics, and Brain histology) including citations of our previous work.
