# Supplementary material for: Pioglitazone restores mitochondrial function but does not spare cortical tissue following mild brain contusion
Source: Brain Commun. 2023 Feb 13;5(2):fcad032. doi: 10.1093/braincomms/fcad032 (PMC9985333; doi:10.1093/braincomms/fcad032)
Supplement: fcad032_Supplementary_Data [file Revision 1 - Decision letter.pdf › Revision 1 - Decision letter.pdf]

Revision 1 – Decision Letter

22-Nov-2022

Dear Dr. Sullivan,

Thank you for your recent submission BRAINCOM-2022-317.R1
entitled "Pioglitazone restores mitochondrial function but does not spare cortical tissue following mild brain contusion" to Brain Communications.

I am pleased to let you know that we would like to provisionally accept your manuscript entitled "Pioglitazone restores mitochondrial function but does not spare cortical tissue following mild brain contusion" for publication in Brain Communications. There are still some minor points for you to address before final acceptance, but further peer-review will not be necessary.
Please would you respond to the comments shown below from reviewers and/or our Scientific Editor (regarding our requirements for presentation of scientific data at Brain Communications) and submit a revised version.

Please format your revised manuscript according to our Instructions to Authors https://academic.oup.com/braincomms/pages/General_Instructions – a summary of key points is attached to help with this.

To revise your manuscript, click the link below or log into https://mc.manuscriptcentral.com/braincom and enter your Author Centre, where you will find your manuscript title listed under "Manuscripts with Decisions." Under "Actions," click on "Create a Revision." Your manuscript number will be appended to denote a revision.

Please upload a revised manuscript with the changes you have made shown either with "track changes" or in coloured text.

When submitting your revised manuscript, you will be asked to respond to the reviewer(s) and editor's comments. Please be as specific as possible in your response to all of the comments.

The editorial team at Brain Communications also aims to increase transparency in scientific publishing and to demonstrate the value that peer review brings to the scientific process. Please therefore consider whether you are willing to have reviewer comments on your paper and your response published as supplementary data with the paper if it is accepted. This is entirely optional and will only be done if both the authors and reviewers agree. When submitting your revision you will have the opportunity to update the answer you entered previously.

IMPORTANT: Your original files are available to you when you upload your revised manuscript. Please delete any redundant files before completing the submission.

Because we are trying to facilitate timely publication of manuscripts submitted to Brain Communications, we would appreciate your revised manuscript as soon as possible. If it is not possible for you to submit your revision within 3 months, the option to submit a revision will expire, and we will consider your revised paper as a new submission. However, if you need a little more time, please get in touch and we can extend this deadline for you.

Once again, thank you for submitting your manuscript to Brain Communications and I look forward to receiving your revision.

Best wishes

Ms. Elizabeth Simzer
Scientific Editor, Brain Communications

Professor Tara Spires-Jones
Founding Editor, Brain Communications

Reviewer(s)' Comments to Author:

Reviewer: 1 (Michela Marcatti)

Comments to the Author
The revised manuscript is well improved. The authors addressed all my concerns so now the manuscript is suitable for publication in Brain Communications.
Thank you

Reviewer: 2 (Janna Harris)

Comments to the Author
An attentive revision has significantly clarified the results and enhanced the rigor of the manuscript. It is important to study promising therapies across a range of preclinical TBI models and severities, and the authors are commended for their contribution.

Comments from the Scientific Editorial Team:

Please see the attached iThenticate report. The iThenticate report system has flagged some portions of the Materials and Methods. Please, replace them with the citation of the paper or rephrase them. The parts we are referring to are:
- Total mitochondrial isolation
- Measurement of mitochondrial bioenergetics
- Brain histology
